# Patient-derived castration-resistant prostate cancer model revealed CTBP2 upregulation mediated by OCT1 and androgen receptor

**DOI:** 10.1186/s12885-024-12298-3

**Published:** 2024-05-02

**Authors:** Daisuke Obinata, Kenichi Takayama, Mitchell G Lawrence, Daigo Funakoshi, Makoto Hara, Birunthi Niranjan, Linda Teng, Renea A Taylor, Gail P Risbridger, Satoru Takahashi, Satoshi Inoue

**Affiliations:** 1 Department of Urology, Nihon University School of Medicine, 30-1, Ooyaguchikamicho, Itabashi-ku, Tokyo, 173-8610 Japan; 2 Monash Biomedicine Discovery Institute, Department of Anatomy and Developmental Biology, Monash University, Wellington Road, Clayton, VIC 3800 Australia; 3 Department of Systems Aging Science and Medicine, Tokyo Metropolitan Institute for Geriatrics and Gerontology, 35-2 Sakae-cho, Itabashi-ku Tokyo, Tokyo, 173-0015 Japan; 4 Cancer Research Division, Peter MacCallum Cancer Centre, 305 Grattan Street, Parkville, VIC 3000 Australia; 5 Sir Peter MacCallum Department of Oncology, The University of Melbourne, 305 Grattan Street, Parkville, VIC 3010 Australia; 6 Cabrini Institute, Cabrini Health, 183 Wattletree Road, Malvern, VIC 3144 Australia; 7 Division of Neurology, Department of Medicine, Nihon University School of Medicine, 30-1, Ooyaguchikamicho, Itabashi-ku, Tokyo, 173-8610 Japan; 8 Monash Biomedicine Discovery Institute, Department of Physiology, Monash University, Wellington Road, Clayton, VIC 3800 Australia; 9 Research Center for Genomic Medicine, Saitama Medical University, 1397-1 Yamane, Hidaka-shi, Saitama, 350-1241 Japan

**Keywords:** Androgen receptor, OCT1, Prostate cancer, CTBP2

## Abstract

**Background:**

Prostate cancer is dependent on androgen receptor (AR) signaling, and androgen deprivation therapy (ADT) has proven effective in targeting prostate cancer. However, castration-resistant prostate cancer (CRPC) eventually emerges. AR signaling inhibitors (ARSI) have been also used, but resistance to these agents develops due to genetic *AR* alterations and epigenetic dysregulation.

**Methods:**

In this study, we investigated the role of OCT1, a member of the OCT family, in an AR-positive CRPC patient-derived xenograft established from a patient with resistance to ARSI and chemotherapy. We conducted a genome-wide analysis chromatin immunoprecipitation followed by sequencing and bioinformatic analyses using public database.

**Results:**

Genome-wide analysis of OCT1 target genes in PDX 201.1 A revealed distinct OCT1 binding sites compared to treatment-naïve cells. Bioinformatic analyses revealed that OCT1-regulated genes were associated with cell migration and immune system regulation. In particular, *C-terminal Binding Protein 2 (CTBP2)*, an OCT1/AR target gene, was correlated with poor prognosis and immunosuppressive effects in the tumor microenvironment. Metascape revealed that CTBP2 knockdown affects genes related to the immune response to bacteria. Furthermore, TISIDB analysis suggested the relationship between CTBP2 expression and immune cell infiltration in prostate cancer, suggesting that it may contribute to immune evasion in CRPC.

**Conclusions:**

Our findings shed light on the genome-wide network of OCT1 and AR in AR-positive CRPC and highlight the potential role of *CTBP2* in immune response and tumor progression. Targeting *CTBP2* may represent a promising therapeutic approach for aggressive AR-positive CRPC. Further validation will be required to explore novel therapeutic strategies for CRPC management.

**Supplementary Information:**

The online version contains supplementary material available at 10.1186/s12885-024-12298-3.

## Background

Androgens and the androgen receptor (AR) are crucial in regulating the growth and progression of prostate cancer, hence androgen deprivation therapy (ADT) is an effective treatment option for prostate cancer [1]. Yet ADT is only temporarily effective and patients eventually develop castration-resistant prostate cancer (CRPC) [2]. Since AR and its signaling pathways remain abnormally activated even under low androgen conditions in most cases of CRPC, AR signaling inhibitors (ARSI) such as abiraterone acetate, enzalutamide, apalutamide, and darolutamide are effective treatments for CRPC [3–6]. However CRPC cells eventually acquire resistance to continuous treatment with these agents. Common mechanisms underlying this resistance include the expression of AR variants and genetic alterations of the *AR*, such as mutations, amplifications, and structural rearrangements [7]. In addition, recent studies have highlighted the role of transcription factor-mediated epigenetic regulation in CRPC [8]. Dysregulation of transcription factors has been associated with the development of lineage plasticity, leading to the transformation of prostate cancer into AR-independent prostate cancer with neuroendocrine features [9]. 

Among these transcription factors, the octamer transcription factor (OCT) family, including OCT1, has recently gained attention for its impact on prostate cancer progression [10, 11]. Previous reports have shown that the expression patterns of transcription factors that cooperate with AR differ significantly depending on the stage of lineage plasticity-induced phenotypic transition [12, 13]. We previously reported that high OCT1 expression correlates with poor prognosis in prostate cancer patients [14]. Furthermore, a comprehensive analysis of OCT1 signals revealed that Acyl-CoA Synthetase Long-Chain Family Member 3 (ACSL3) was the most highly expressed gene regulated by both AR and OCT1 in hormone-responsive prostate cancer cells (LNCaP cells) [11]. 

Through a genome-wide analysis of OCT1 target genes in AR-positive 22Rv1 cells, we identified a distinct set of target genes from LNCaP cells, including anillin-actin binding protein (ANLN) and discs large homolog-associated protein 5 (DLGAP5), that promotes cell cycle progression and proliferation in prostate cancer cells [15, 16]. These data suggest the possibility that the gene sets targeted by the OCT1/AR complex change during the progression of prostate cancer.

Prostate cancer cell lines such as LNCaP, VCaP and 22Rv1 cells do not fully encompass the characteristics of CRPC heterogeneity and lineage plasticity induced by various treatments. On the other hand, PDXs maintain the histological and genomic characteristics of the original patient tumors, and provide a more effective research model to study these concepts [8, 17]. In a previous study, we established novel PDXs of CRPC resistant to conventional ADT, second-generation AR-targeted inhibitors, and chemotherapy, including AR-positive CRPC models [18]. PDX 201.1 A is a patient-derived model of AR-positive CRPC that was established from a rapid autopsy sample of a dura metastasis from a prostate cancer patient [18]. In this study, we used PDX 201.1 A to elucidate the genome-wide network of OCT1 in AR-positive CRPC induced by actual ARSIs and taxane-based chemotherapies.

## Methods

### Cell culture and reagents

The patient treatment history of PDX 201.1 A is was previously described [18]. The patient demonstrated resistance to ARSI agents and taxane-based chemotherapy, ultimately leading to prostate cancer-specific death. To produce in vitro cultures, PDX 201.1 A was minced and grown in advanced DMEM/F-12 media containing 1% penicillin-streptomycin, 2 mM Glutamax, 1 nM DHT, 1.25 mM N-acetylcysteine, 50 ng/ml EGF, 500 nM A83-01, 10 mM, nicotinamide, 10 µM SB202190, 2% B27, 100 ng/ml noggin, 10 ng/ml FGF10, 5 ng/ml FGF2, 1 µM prostaglandin E2 and 10% R-spondin 1 conditioned media. 10 µM Y-27,632 dihydrochloride as previously described [18]. 

### Immunohistochemistry

Immunohistochemistry was performed on PDX201.1 A tissue using the Leica BOND-MAX-TM automated system (Autostainer) with BondTM epitope retrieval (ER)-1 for anti-AR (Sigma) and ER-2 for anti-OCT1 (Abcam) antibody [18, 19]. 

### Chromatin immunoprecipitation and chromatin immunoprecipitation-sequencing (ChIP and ChIP-seq)

201.1 A cells were treated with vehicle or 10 nM of DHT for 24 h. AR/OCT1 ChIP and quantitative PCR (qPCR) were performed as previously described [11, 20]. The fold enrichment relative to the IgG-IP control or input was quantified qPCR using Power SYBR™ Green PCR Master Mix (Applied Biosystems™) and the Stratagene Mx3000P (Agilent Technologies). The primer sequences for the detection of ARBSs by qPCR are listed below.

ACSL3 enhancer Fw: 5’-TCCTGCTGTACTCATTGTTACTAGAATAAA-3’

Rv: 5’-GCTTTTCATTTGTCAGAGTGCTAAGTAT-3’.

AR, OCT1, and Acetyl-Histone H3 (Lys27) (AcH3K27) ChIP-seq from PDX 201.1 A cells were performed as in previous studies using an Illumina HiSeq 2500 (Illumina, San Diego, CA, USA) [16]. The signal score for AR and OCT1 bindings were calculated using Model-based analysis of ChIP-seq (MACS; 20). The threshold for binding sites was set at *p* < 1.0e-4. The dataset was examined using the Integrative Genome Viewer. Genes located within 1000 kb of the upstream and downstream of the binding site were extracted with the Genomic Regions Enrichment of Annotations Tool (GREAT) version 4.0.4 to be candidates for AR and OCT1-target genes [21]. Motif search (50 bp around the peaks obtained by ChIP-seq) was performed using HOMER [22], and the samples used for ChIP-seq were further validated for binding to some regions by qPCR as described below. To identify putative super-enhancers, we used Rank Ordering of Super-enhancers (ROSE) downloaded from Young Lab (http://younglab.wi.mit.edu/super_enhancer_code.html) [23]. 

### Analysis of clinical data in the public databases

We compared the transcriptional expression of *AKAP12, CTBP2, HIF1A* and *RHOB*, and prognosis of prostate cancer cases based on publicly available data from Gene Expression Profiling Interactive Analysis (GEPIA) [24]. In addition, differential expression levels of *CTBP2* were analyzed using two datasets (GSE35988 and GSE3325) downloaded from the Gene Expression Omnibus (GEO). The p value was determined by the Kruskal-Wallis test. Using data from the Cancer Genome Atlas database, GEPIA performed the Log-rank test to analyze overall survival (OS) based on gene expression. To correlate the abundance of tumor-infiltrating lymphocytes (TILs) with the expression level of CTBP2 in prostate cancer, we utilized TISIDB (http://cis.hku.hk/TISIDB/index.php). TISIDB is a web portal that focuses on tumor-immune interactions and integrates multiple heterogeneous datasets [25]. 

### Analysis of expression changes in immune checkpoint-related genes

The gene clusters for immune checkpoints were selected based on previously reported papers [26, 27]. Microarray results of prostate cancer cell line LNCaP cells treated with either CTBP2-suppressing siRNA or control siRNA were obtained from our previous report [28]. 

## Results

### Identification of OCT1 and AR expression and binding sites in AR-positive CRPC with previous ARSI treatment

**Fig. 1:**
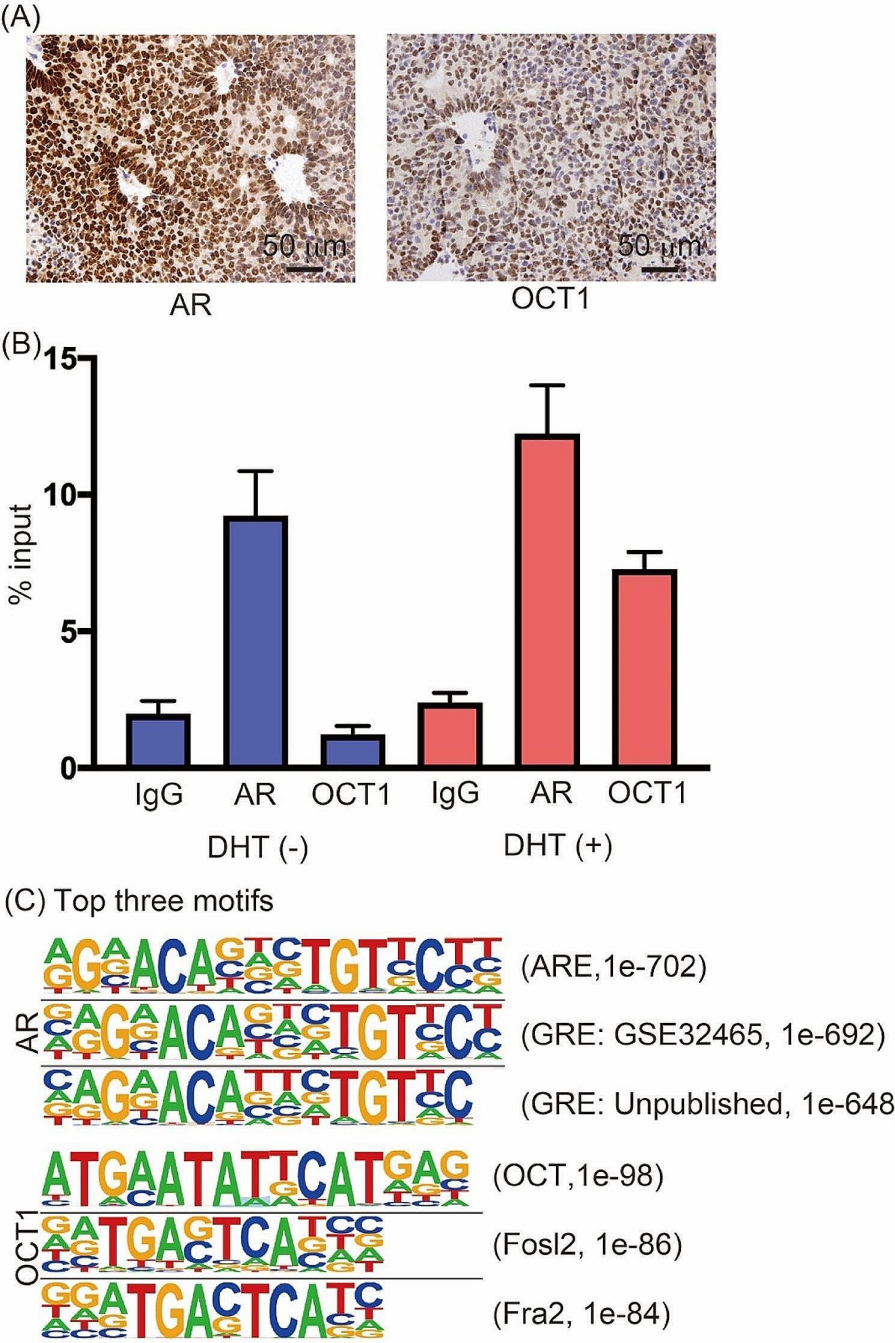
Analysis of octamer transcription factor (OCT1) expression and binding in androgen receptor (AR)-positive castration-resistant prostate cancer (CRPC) patient-derived xenograft (PDX). (**A**) Representative images of immunohistochemistry for AR and OCT1 in PDX 201.1 A. Scale bars equal 50 μm. (**B**) Chromatin immunoprecipitation (ChIP) analysis of AR and OCT1 in ACSL3 enhancer region representative AR-binding sites (ARBSs) in PDX201.1 A cells. Enrichment of AR and OCT1 within ACSL3 enhancer region were quantified by quantitative polymerase chain reaction (qPCR). (**C**) Motif analysis of AR and OCT1-binding regions PDX201.1 A cells showing the enrichment of ARE and OCT, respectively. We used HOMER motif analysis for 200-bp DNA sequences around AR and OCT1-binding peaks. The top motifs by this analysis are related to AR and OCT respectively

We previously reported that OCT1 is recruited in an androgen-dependent manner and that its targeting genes differ between cells [11, 16]. However, these studies were performed in treatment naïve cells, that had not been exposed to ARSI treatment or acquired therapy resistance. Hence, we examined AR and OCT1 expression in the AR-positive CRPC tumor, PDX 201.1 A. Using immunohistochemistry, we showed that AR and OCT1 were both strongly expressed in the nucleus of PDX 201.1 A (Fig. 1A). Then, we cultured cells from PDX 201.1 A in vitro and treated them with dihydrotestosterone (DHT) to confirm the androgen dependent recruitment of AR and Oct1 to AR binding sites (ARBSs) in the enhancer region of *ACSL3* [11], a representative target gene (Fig. 1B). ChIP-seq analysis was performed to identify genome-wide activated histone markers, AcH3K27 sites, AR, and OCT1 binding regions in PDX 201.1 A (GEO repository (www.ncbi.nlm.nih.gov/geo), accession number GSE193073). The HOMER program showed that the top three motifs for AR ChIP-seq related to steroid hormone family‒binding motifs, while the top motifs for OCT1 ChIP-seq were for OCT binding (Fig. 1C). This data confirms the enrichment of regions with direct AR and OCT1 binding sites.

### Combined analysis of hyperacetylated chromatin domains with AR and OCT1-binding sites and RNA sequencing (RNA-seq) data identified putative AR and OCT1-regulated genes in AR-positive CRPC

**Fig. 2:**
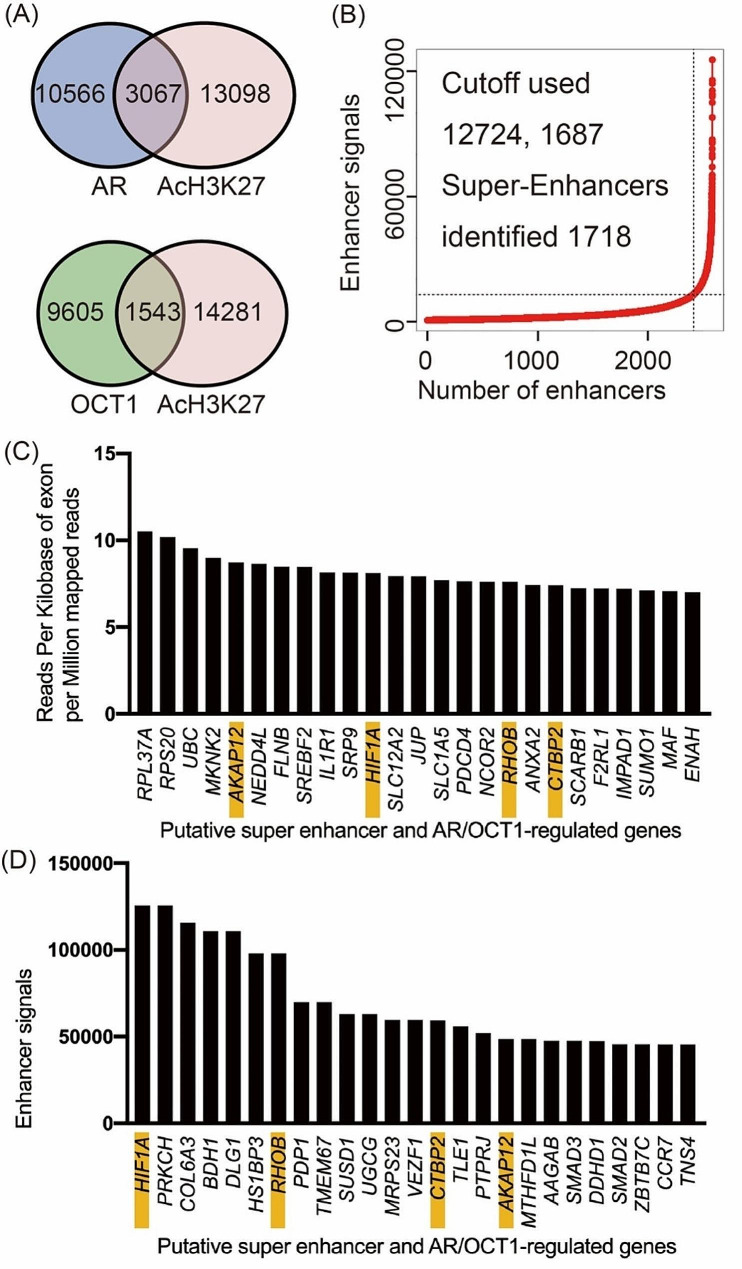
Identification of Acetyl-Histone H3 (Lys27) (AcH3K27), AR, and OCT1-binding regions by chromatin immunoprecipitation-sequencing (ChIP-seq). (**A**) ChIP-seq analyses were performed using PDX-201.1 A cells. AcH3K27, AR, and OCT1-binding regions (vs. input control, *p* < 1.0e-4) were determined by model-based analysis for ChIP-seq (MACS). (**B**) Line graph showing the number of putative super-enhancers defined by ranked AcH3K27 signal. (**C**) Top twenty-five highly expressed genes among the putative super-enhancer regulated genes in the vicinity of common AR and Oct1-binding sites in PDX 201.1 A cells. (**D**) Top twenty-five genes with highly enhancer signals among the putative super-enhancer regulated genes in the vicinity of common AR and Oct1-binding sites in PDX 201.1 A cells

AR and OCT1-binding regions and AcH3K27 enriched sites were detected by calculating the enrichment compared with the input using MACS software. In total, 13,633 binding regions and 16,165 regions were identified as AR and AcH3K27 enriched sites, 9,605 binding regions and 15,824 regions were identified as OCT1 and AcH3K27 enriched sites (vs. input control, *p* < 1.0e-4) (Fig. 2A). Of these, 3067 regions overlapped between AR and Ach3K27, and 1543 regions overlapped between OCT1 and AcH3K27 (Fig. 2A). Based on the AcH3K27ChIP-seq data, ROSE analysis [23] identified 1718 putative super-enhancers (Fig. 2B). We also identified a common site among OCT1, AR, and putative super-enhancers and used GREAT version 4.0.4 to detect 221 putative AR and OCT1-regulated genes located within 1000 kb of the site. Using our previous RNA-seq data, we examined the mRNA levels of these genes [18]. The top 25 genes based on high expression (Fig. 2C) and enhancer signal levels (Fig. 2D) are shown for putative super-enhancer, AR, and OCT1-regulated genes. Several genes were present in both categories, including hypoxia inducible factor 1 subunit alpha (*HIF1A)*, ras homolog family member B *(RHOB)*, C-terminal binding protein 2 *(CTBP2)*, and A-kinase anchoring protein 12 (*AKAP12)*. To validate these results, we examined additional RNA-seq data to compare the expression of candidate transcripts in 201.1 A to the AR-positive LNCaP cell line and eleven additional PDXs of AR positive CRPC [29]. These findings indicated that, in addition to OCT1, three of the four focused genes, with the exception of RHOB, were more highly expressed in PDX models than in LNCaP, particularly in PDX201.1 (Additional file 1).

### Identification of AR and OCT1-regulated genes that mediate cell migration

**Fig. 3:**
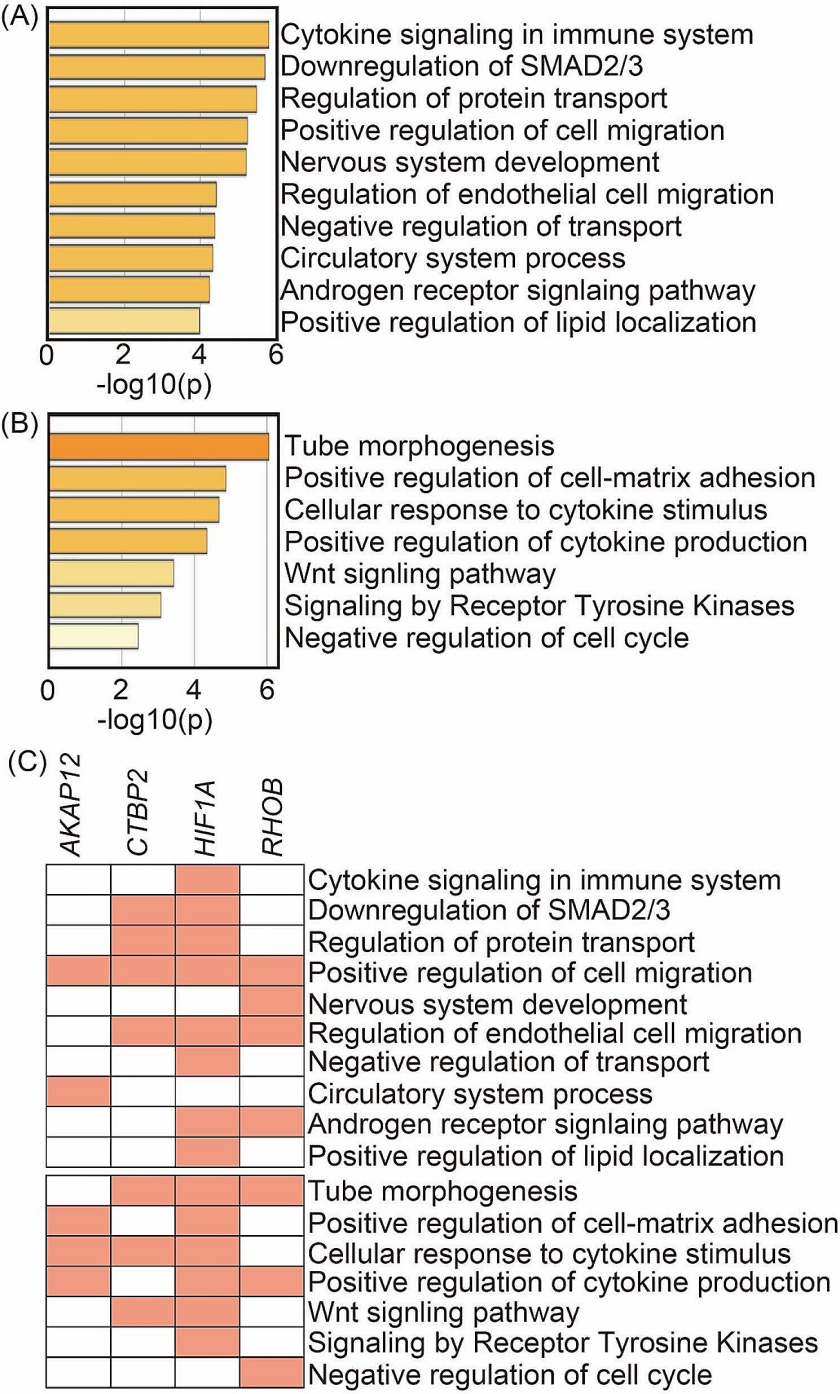
Identification of putative AR/OCT1-regulated key genes. Functional annotations according to the top 25 highest expression (**A**) and enhancer signals (**B**) for putative AR/OCT1-regulated genes in PDX 201.1 A cells. (**C**) List of biological functions showing unique and common features of putative super-enhancer regulated genes in the vicinity of AR/OCT1-binding sites in PDX 201.1 A cells

To examine the relationship between AR and OCT1 binding and super-enhancer-regulated gene function, a web-based analysis tool, Metascape, was used to find common biological functions in the selected gene list [30]. We extracted a list of the top 25 most highly expressed genes among putative super-enhancer, AR, and OCT1-regulated genes. These genes were associated significantly enriched in cytokine signaling in immune system, downregulation of SMAD2/3, regulation of protein transport, positive regulation of cell migration, and nervous system development (Fig. 3A). We also extracted the top 25 genes with high enhancer signals and they were enriched for tube morphogenesis, positive regulation of cell-matrix adhesion, and cellular response to cytokine stimulus (Fig. 3B). Notably, in both settings, there was enrichment of a group of genes that appear to promote migration. Thus, we examined whether four genes (*AKAP12*, *CTBP2*, *HIF1A* and *RHOB*) that match both categories enriched these annotations. Interestingly, all of 4 genes enriched the annotation of positive regulation of cell migration (Fig. 3C).

### CTBP2 correlates with poor prognosis of prostate cancer and immune infiltration

**Fig. 4:**
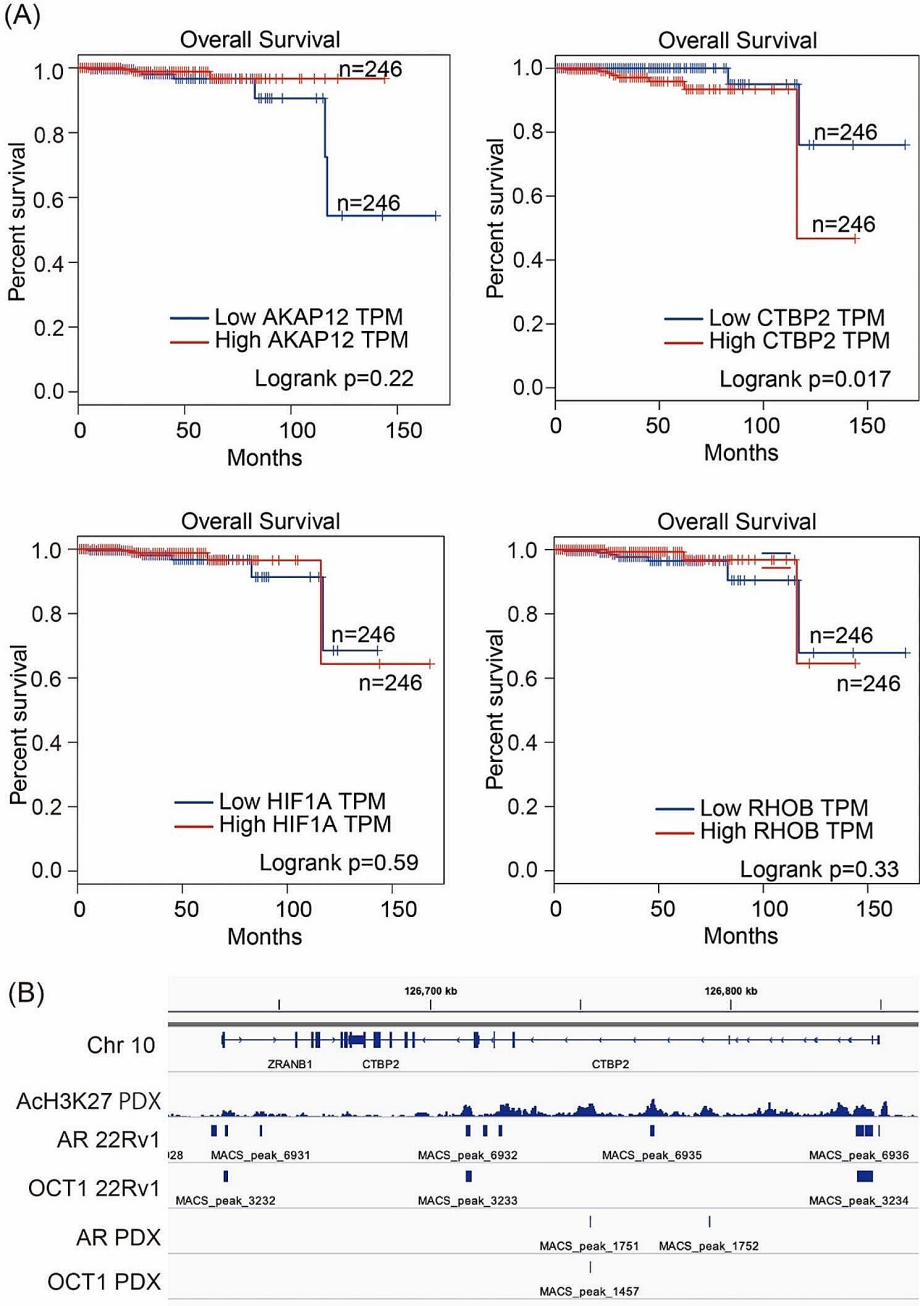
High expression levels of putative AR/OCT1-regulated key genes in prostate tumors are correlated with poor prognosis. (**A**) We analyzed the overall survival of patients by using GEPIA. The cut off was set at 50% for high expression and 50% for low of 492 tumors. P value was calculated by log rank test. (**B**) Identification of AcH3K27, ARBS and OCT1 binding cites around the CTBP2 gene in PDX201.1 A cells and comparison with ARBS and OCT1 in 22Rv1 cells

To evaluate the correlation between these 4 genes and overall survival of prostate cancer, we used GEPIA, an online tool for analyzing RNA-seq expression data [24]. Using the Cancer Genome Atlas (TCGA) dataset, we observed that high expression of *CTBP2* was significantly correlated with poor overall survival of prostate cancer patients (Fig. 4A), while *AKAP12, HIF1A* and *RHOB* were not. Furthermore, the expression levels of *CTBP2* among the three groups of benign, primary, and metastatic CRPC tissues were compared. *CTBP2* expression was significantly elevated in metastatic CRPC tissues compared to that in benign and primary prostate cancer tissues (Additional file 2). Binding sites for ARBS and OCT1 were found in the intron and 5’ upstream region of *CTBP2*, similar to previous ChIP-seq data from 22Rv1 (Fig. 4B) [16]. 

**Fig. 5:**
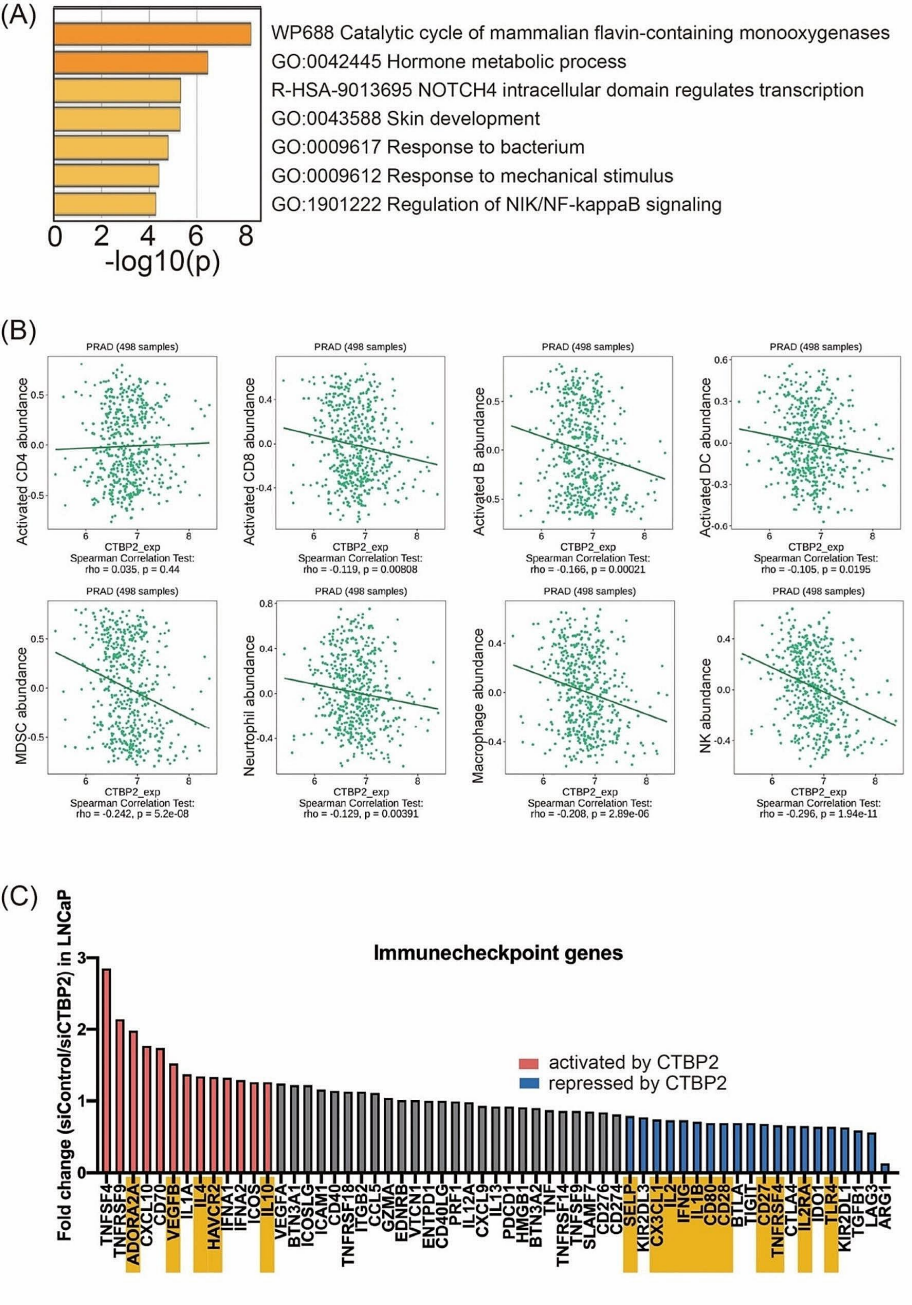
Correlation between *CTBP2* expression and tumor-infiltrating lymphocytes (TILs) in prostate cancer. (**A**) Functional annotations for the top 200 decreased genes by *CTBP2* in previously published microarray data using LNCaP cells. (**B**) The association between *CTBP2* expression levels and immune cell infiltration into the tumor was assessed using TISIDB database. Spearman correlation coefficients (Rho) and corresponding p values are presented to indicate the strength and significance of the correlation. CD4; CD4 T lymphocytes, CD8; CD8 T lymphocytes, B; B lymphocytes, DC; dendritic dell, MDSC; myeloid-derived suppressor cells, NK; natural killer cells. (**C**) CTBP2-induced changes in expression of immune checkpoint-related genes, based on our previous microarray data [28] using LNCaP cells treated with siRNA suppressing CTBP2 (siCTBP2) or control (siControl), compared to siCTBP2. Genes increased by more than 1.25-fold in siControl, and those decreased by less than 0.8-fold are colored

Using Metascape, we found that the genes decreased by *CTBP2* knockdown are associated with several gene ontology categories including immune response to bacterium (Fig. 5A). Then, we employed TISIDB (an integrated repository portal for tumor-immune system interactions) to investigate the relationship between *CTBP2* expression and the infiltration of immune cells in prostate cancer. The analysis revealed a significant association between *CTBP2* levels and immune cell infiltration within the tumor microenvironment (Fig. 5B). Specifically, higher *CTBP2* expression levels were negatively correlated with the presence of activated B cells (rho = -0.166), activated CD8 + T cells (rho = -0.119), macrophages (rho = -0.208), dendritic cells (rho = -0.105), myeloid-derived suppressor cells (MDSC; rho = -0.242), neutrophils (rho = -0.129), and natural killer cells (rho = -0.296) (Fig. 5B). On the other hand, no significant correlation was observed between *CTBP2* expression and the infiltration of activated CD4 + T cells (rho = 0.035) (Fig. 5B). Next, we extracted a list of previously reported immune checkpoint-related genes [26, 27] from the microarray data on LNCaP cells in which CTBP2 expression was knocked down with siRNA [28]. The microarray study found that immune checkpoints genes affecting TILs, such as *ADORA2A* and *CD80*, are regulated by CTBP2 (Fig. 5C).

## Discussion

AR plays a crucial role in prostate cancer and is also important in the development of CRPC. ASRIs and taxane-based chemotherapeutic agents are used to treat CRPC, however CRPC cells adapt to these therapies through genetic alterations of the *AR* and dysregulation of transcription factor networks [9]. Notably, it has been recently reported that abnormalities in the *AR* gene are the dominant genomic driver for acquired treatment-resistance against CRPC treatments [7], although epigenetic modifications are widely affected. Therefore, it is expected that abnormalities in other transcription factors without genetic alterations would have a key role in such epigenetic changes during the progression of CRPC.

The utility of PDXs has greatly enhanced the capacity to study the diversity of human prostate cancer, including the emergence of CRPC. In a previous study, we established four PDXs from rapid autopsy samples of two CRPC patients who had exhausted multiple treatments, including one AR-negative CRPC model [18]. Furthermore, we identified that *OCT1* is highly expressed in that AR-negative CRPC-PDX (PDX 201.2), and *OCT1* is involved in the AR-independent expression of genes that promote cell migration and tumor proliferation [19]. Most AR-responsive genes require AR transcriptional coactivators that act on AR binding regions to support AR binding and subsequently express androgen-responsive genes. Among these transcription factors, GATA-binding protein 2 (*GATA2*), Forkhead box A1 (*FOXA1*), and *OCT1* are significantly enriched with functional AR in 68% of AR binding regions [31]. However, the distribution of AR binding regions and the types of cooperating transcription factors differ between hormone-sensitive prostate cancer and CRPC. CRPC-specific AR binding regions are not primarily occupied by typical AR-cooperating factors observed in hormone-sensitive prostate cancer cells but rather by AR-independent transcription factors, such as MYC [13]. 

In this study, we identified *CTBP2* as a candidate AR/OCT1 target gene and showed that it is associated with prognosis of prostate cancer. We previously reported that *CTBP2* not only acts as an AR responsive gene, but also it regulates the activity of AR as a co-regulator [28]. High *CTBP2* expression levels correlate with poor prognosis in prostate cancer patients, while CTBP2 suppresses cancer-inhibiting genes and AR inhibitory factors, such as NCOR and RIP140, by binding to their transcription regulatory regions in prostate cancer cells [28]. On the other hand, CTBP1/2 are known to function as NADH-dependent transcriptional repressors [32]. Cancer cells prefer anaerobic glycolysis (Warburg effect) to maintain rapid proliferation, resulting in elevated levels of NADH [33]. In breast cancer, CTBP1/2 acts as an epigenetic effector downstream of elevated NADH [33], suggesting that CTBP2 is involved in promoting energy production in cancer cells.

Prostate cancer exists in an immunosuppressive microenvironment [34]. The presence of TILs is generally associated with improved patient outcomes in many cancer types [35]. The prostate cancer tumor microenvironment (TME) contains various subtypes of immune-suppressive cells, such as MDSCs and tumor-associated macrophages (TAMs), which inhibit the functions of immune effector cells. MDSCs have been identified as important factors in constructing an immunosuppressive and tumor-promoting TME in prostate cancer [36]. Additionally, TAMs, as major components of the TME, play a crucial role in promoting matrix remodeling and angiogenesis while inhibiting cytotoxicity and antigen-presenting abilities, contributing to immune suppression and influencing the progression of many cancers [37]. Moreover, enzalutamide-resistant prostate cancer cells have been reported to become immunosuppressed during the transition to the resistant stage. Enhancing the anti-tumor activity of immune cells involves increasing T cell activation and cytotoxic T cell killing activity, while simultaneously suppressing Tregs and MDSCs. This approach can result in reduced depletion of TILs and more effective tumor control [34]. 

In the present study, we observed a negative correlation between *CTBP2* expression in prostate cancer and MDSCs and TAMs, which are known to promote tumor progression. Interestingly, *CTBP2* expression also exhibited a negative correlation with activated TILs, which are known to play crucial roles in inhibiting cancer through immune responses. On the other hand, the immune checkpoint-related genes affect TILs and have an impact on the prognosis of various cancers. By using microarray data, we observed a potential association between CTBP2 and the regulation of expression levels of these genes in prostate cancer cells. According to a previous report [27], five (*ADORA2A, VEGFB, IL4, HAVCR2*, and *IL10*) of the 13 immune checkpoint-related genes upregulated by CTBP2, were inhibitory, and 11 (*SELP, CX3CL1, IL2, IFNG, IL1B, CD80, CD28, CD27, TNRSF4, IL2RA*, and *TLR4*) of the 20 genes downregulated by CTBP2, were stimulatory. In particular, *ADORA2A* mediates immunosuppression in TME and has been suggested to be associated with prostate cancer prognosis [38]. Conversely, *CD80* is an M1 macrophage marker and decreased in enzalutamide-resistant prostate cancer which is directly promoted by immunosuppressive signaling [34]. Furthermore, the expression pattern of this group of genes approximated C5 (Immunologically Quiet) of the six immune subtypes [27]. Collectively, these findings suggest that *CTBP2* may exert an overall immunosuppressive effect, thereby favoring tumor growth in the prostate cancer microenvironment. Although it is unclear how *CTBP2*, which is related to AR-responsive genes and AR sensitivity, affects the immune system, our study is the first to report that OCT1/AR target genes located in super-enhancer regions induced by treatment have an impact on the immune system. This study had several limitations. PDX models are highly useful in preclinical research as they broadly represent the diversity among patients. However, assays are more challenging to perform with patient-derived cells compared to immortalized cell lines. Our previous experiments showed that knockdown of CTBP2 in prostate cancer cell lines affects their proliferation and migration abilities [28]. Although, these results suggested that CTBP2 may promote the proliferation and migration of PDX201.1, further studies will be required to directly evaluate the impact of CTBP2 knockdown on cell migration in PDX201.1.

## Conclusions

In conclusion, our study revealed the global OCT1 binding sites in an AR-positive patient-derived model of CRPC. The OCT1 targets were significantly enriched for cell migration and microtubule pathways. We also identified a potential association between *CTBP2* the prognosis of prostate cancer, as well as its potential influence on TILs. While further in vitro experiments are necessary to validate our findings, we propose that targeting CTBP2 could be a promising therapeutic strategy for aggressive treatment-resistant AR-positive CRPC.

### Electronic supplementary material

Below is the link to the electronic supplementary material.

Additional file 1: Comparison of gene expression levels in LNCaP, 201.1A and 11 other PDXs of AR-positive CRPC

Additional file 2: CTBP2 Gene Expression in benign, primary, and mCRPC tissues

## Data Availability

The RNA-seq dataset has previously been reported [18] and is available on request from the MURAL. ChIP-seq data has been deposited in the GEO repository (www.ncbi.nlm.nih.gov/geo), accession number GSE193073. The remaining datasets generated during and/or analyzed during the current study are available from the corresponding author on reasonable request.

## Abbreviations

ACH3K27: Histone h3 lysine (k)27 acetylation
ACSL3: Acyl-coa synthetase long-chain family member 3
ADT: Androgen deprivation therapy
AKAP12: A-kinase anchoring protein 12
ANLN: Anillin-actin binding protein
AR: Androgen receptor
ARBS: AR binding sites
ARSI: Androgen receptor signaling inhibitor
ChIP-seq: Chromatin immunoprecipitation and chromatin immunoprecipitation-sequencing
CRPC: Castration-resistant prostate cancer
CTBP2: C-terminal binding protein 2
DHT: Dihydrotestosterone
DLGAP5: Discs large homolog-associated protein 5
ER: Epitope retrieval
FOXA1: Forkhead box a1
GATA2: GATA-binding protein 2
GEO: Gene expression omnibus
GEPIA: Gene expression profiling interactive analysis
GREAT: Genomic regions enrichment of annotations tool
HIF1A: Hypoxia inducible factor 1 subunit alpha
MACS: Model-based analysis of ChIP-seq
MDSCs: Myeloid-derived suppressor cells
MURAL: Melbourne urological research alliance
OCT: Octamer transcription factor
OS: Overall survival
PDX: Patient-derived xenograft
qPCR: Quantitative PCR
RHOB: Ras homolog family member b
RNA-seq: RNA sequencing
ROSE: Rank ordering of super-enhancers
TAMs: Tumor-associated macrophages
TCGA: The cancer genome atlas
TILs: Tumor-infiltrating lymphocytes
TME: Tumor microenvironment
