# Supplementary figures and images for: Patient-derived castration-resistant prostate cancer model revealed CTBP2 upregulation mediated by OCT1 and androgen receptor
Source: BMC Cancer. 2024 May 2;24:554. doi: 10.1186/s12885-024-12298-3 (PMC11067191; doi:10.1186/s12885-024-12298-3)

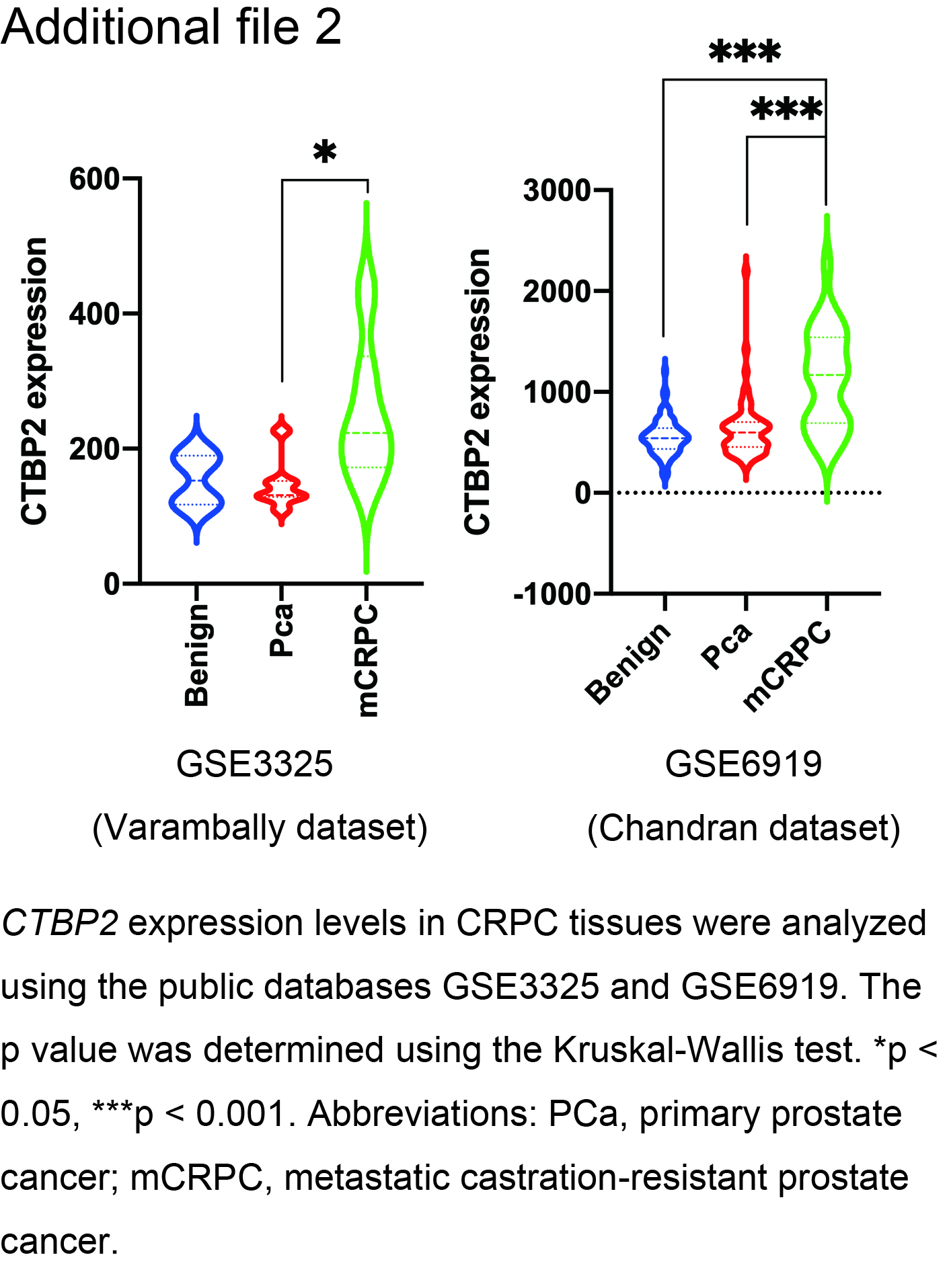

Supplement: Supplementary file 1 — Additional file 1: Comparison of gene expression levels in LNCaP, 201.1A and 11 other PDXs of AR-positive CRPC [file 12885_2024_12298_MOESM1_ESM.jpg]

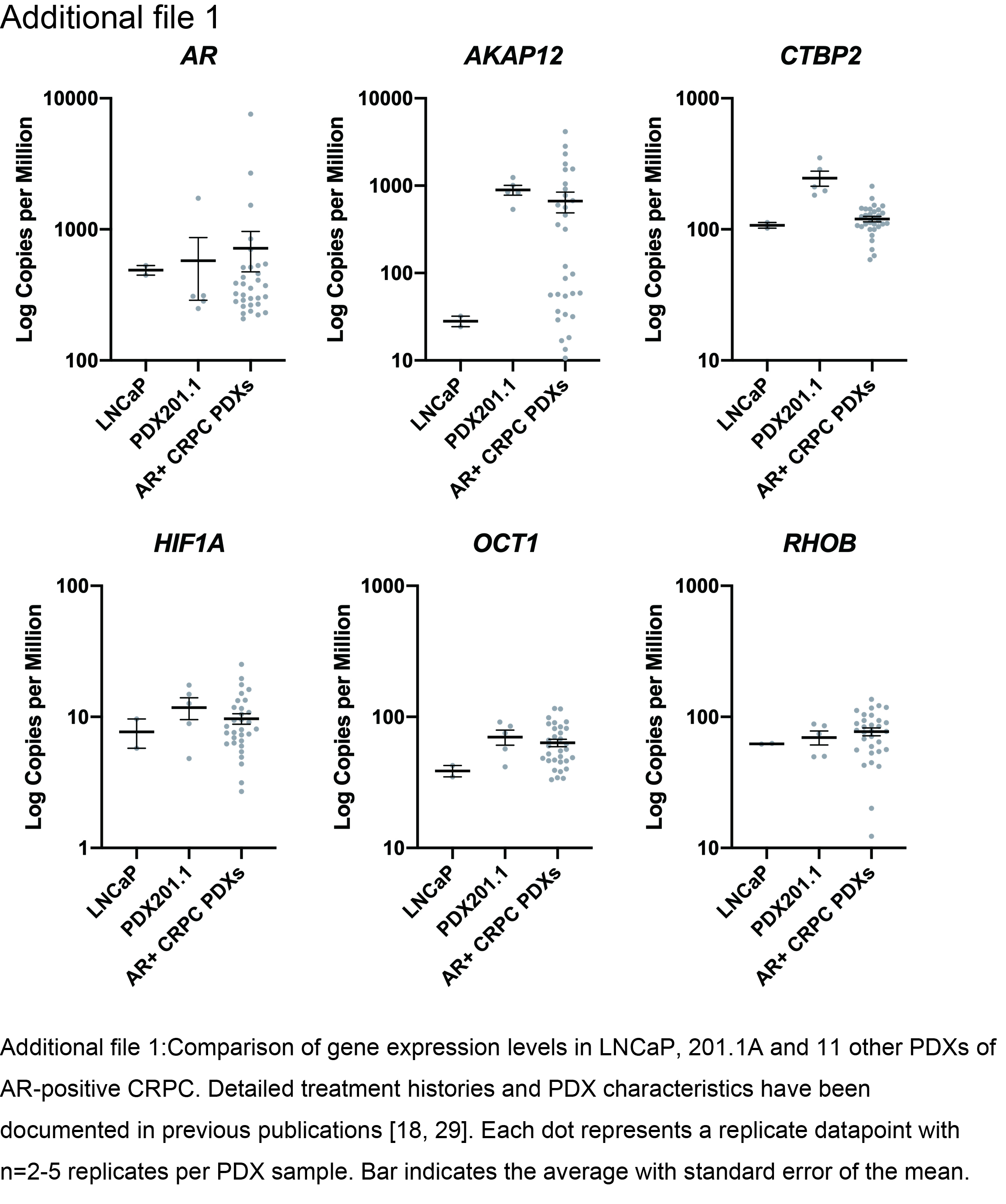

Supplement: Supplementary file 2 — Additional file 2: CTBP2 Gene Expression in benign, primary, and mCRPC tissues [file 12885_2024_12298_MOESM2_ESM.jpg]
